# Supplementary material for: Impact of providing free HIV self-testing kits on frequency of testing among men who have sex with men and their sexual partners in China: A randomized controlled trial
Source: PLoS Med. 2020 Oct 9;17(10):e1003365. doi: 10.1371/journal.pmed.1003365 (PMC7546488; doi:10.1371/journal.pmed.1003365)
Supplement: Attachment — Submitted filename: zhang.pdf [file pmed.1003365.s002.pdf]

This paper presents a clinical trial of the use of self–testing kits for HIV by men who have sex with men in China.

I was asked for a statistical report and I interpret that to include all aspects of the design and conduct of the study.

Points of detail

Page 3 It would be better to present confidence intervals here for the differences, not just p–values. This also applies elsewhere in the paper.

Page 11 The allocation procedure presented here is not the gold standard way of doing this. Using blocks of size two is too small and using a fixed block size makes it too easy to guess the next allocation at some point. It would have been better to use sliding block sizes. Doing the whole thing with sealed envelopes is also open to possible abuse. Obviously this cannot be changed now but it is not ideal.

Page 15 I am afraid I did not understand the sample size determination at all. It reads as though recruitment happened in two strata: recent testers and non–recent. Was that the plan? Given the standard deviations the outcome cannot feasibly be regarded as Gaussian so what test is being considered here?

Page 15 We need more detail here of the imputation model. It would also be better to cite the package using the journal reference its authors provide. `https://www.jstatsoft.org/v45/i03/` seems to be what is returned by `citation("mice")`.

Page 15 The decision to combine the recent and non–recent testers needs to be justified better than this. The authors have already stated they expected there to be many fewer of them earlier on the page. If they were expected to be different then at least include a two–level factor in all the models to allow for the differences.

Page 16 We really need a list here specifying which confounders were adjusted for. This is not mentioned in the results section as far as I can see.

Page 18 '92% considered themselves homosexual, 26% bisexual, and 2% heterosexual' $92 + 26 + 2 \neq 100$. I suspect 92 should be 72 but the authors need to check this.

Pages 19 to 21, Table 1 It is illogical to test for baseline differences. The null hypothesis is that they were drawn from the same population and any differences are due to chance. But you know for sure that they are due to chance, you randomised them (Senn, 1994).

Page 22, Table 2 Of what is this the median and quartiles? Ideally it should be of all the pairwise differences but that is not specified.

Page 24 Why tell us the total number of partners? Would it not be more meaningful to quote some summary statistic per individual?

Page 26, Table 3 As for Table 2.

Points of more substance

Modelling

The main outcome is a count: number of tests. The natural distribution to consider for counts is the Poisson. Rather than use rank–sum methods the use of a linear model with a log link would have enabled us to obtain easily interpretable coefficients for the ratio between the groups leading to statements that the intervention arm took X% more tests with a confidence interval from L to U. It would also be worth checking for zero–inflation relative to Poisson and over–dispersion. I suppose that an ordered categorical model might also be possible here too. If the authors really want to work on the additive scale then doing a linear model and then using bootstrapping to get the correct confidence intervals might be considered.

Adverse events

If I understand Page 27 correctly the only seroconversions occurred in the intervention arm. There were 11 of them. Using a binomial test this shows a difference at the $p < 0.001$ level so this is worthy of some comment. I would have thought that seroconverting counted as an serious adverse event. I see no mention of the trials steering committee or DSMB/DMEC membership in the paper but if a DMEC of which I was a member had received an interim report tht a serious adverse event was split 6–0 between the arms I think alarm bells would have rung.

Summary

Some more work is needed here.

Michael Dewey

References

S Senn. Testing for baseline imbalance in clinical trials. *Statistics in Medicine*, 13:1715–1726, 1994.
