# Supplementary material for: Impact of providing free HIV self-testing kits on frequency of testing among men who have sex with men and their sexual partners in China: A randomized controlled trial
Source: PLoS Med. 2020 Oct 9;17(10):e1003365. doi: 10.1371/journal.pmed.1003365 (PMC7546488; doi:10.1371/journal.pmed.1003365)
Supplement: Attachment — Submitted filename: zhang2.pdf [file pmed.1003365.s004.pdf]

This paper presents a clinical trial of the use of self–testing kits for HIV by men who have sex with men in China.

I was asked for a statistical report and I interpret that to include all aspects of the design and conduct of the study. When I am asked to look at a re–submission which I had already reviewed on a previous submission I restrict myself on principle to commenting on how the authors have responded to my original comments. I do not review the article again from scratch unless it has been so changed as to amount effectively to a new submission.

Existing comments

I said:

> *I am afraid I did not understand the sample size determination at all. It reads as though recruitment happened in two strata: recent testers and non–recent. Was that the plan? Given the standard deviations the outcome cannot feasibly be regarded as Gaussian so what test is being considered here?*

As far as I can see all the details of the sample size determination have now be removed completely from the manuscript. This is a mistake, the sample size determination is a key part of the reporting of a clinical trial and must be included.

I said:

> *The decision to combine the recent and non–recent testers needs to be justified better than this. The authors have already stated they expected there to be many fewer of them earlier on the page. If they were expected to be different then at least include a two–level factor in all the models to allow for the differences.*

However despite what the authors say in the rebuttal Table 2 makes it clear that sub–group was not included in the model. This is because the authors have used variable selection although this is not reported in the methods section where it should be. Selecting a subset of variables in a way driven by the data leads to a model which is unlikely to replicate (Babyak, 2004). Step–wise methods, if those was what the authors have used, give biased coefficients, goodness of fit is exaggerated, p-values and confidence intervals lose their normal meaning, they have problems in the presence of collinearity, they often fail to replicate on new datasets (although they are rarely tested

in that way) (Copas, 1983; Hurvich and Tsai, 1990; Miller, 1990; Derksen and Keselman, 1992).

The authors need to fit a full model using all the theoretically specified confounders. They should also make sure the methods section clearly specifies what was to be done.

I said:

> *If I understand Page 27 correctly the only seroconversions occurred in the intervention arm. There were 11 of them. Using a binomial test this shows a difference at the* $p < 0.001$ *level so this is worthy of some comment. I would have thought that seroconverting counted as an serious adverse event. I see no mention of the trials steering committee or DSMB/DMEC membership in the paper but if a DMEC of which I was a member had received an interim report that a serious adverse event was split 6–0 between the arms I think alarm bells would have rung.*

The authors in their rebuttal do not feel that seroconversion would be an adverse event. So if everybody in the intervention group had increased their frequency of testing but also seroconverted that would be a success? That seems odd to me.

New point

The authors state that all the data is either in the manuscript or in supplementary files. I did not find it there.

Summary

Some more work is needed here.

Michael Dewey

References

M A Babyak. What you see may not be what you get: a brief, nontechnical introduction to overfitting in regression–type models. *Psychosomatic Medicine*, 66:411–421, 2004.

J B Copas. Regression, prediction and shrinkage (with discussion). *Journal of the Royal Statistical Society — Series B*, 45:311–354, 1983.

S Derksen and H J Keselman. Backward, forward and stepwise automated subset selection algorithms: Frequency of obtaining authentic and noise variables. *British Journal of Mathematical and Statistical Psychology*, 45: 265–282, 1992.

C M Hurvich and C L Tsai. The impact of model selection on inference in linear regression. *American Statistician*, 44:214–217, 1990.

A J Miller. *Subset selection in regression*. Chapman and Hall, London, 1990.
