# Supplementary material for: Impact of providing free HIV self-testing kits on frequency of testing among men who have sex with men and their sexual partners in China: A randomized controlled trial
Source: PLoS Med. 2020 Oct 9;17(10):e1003365. doi: 10.1371/journal.pmed.1003365 (PMC7546488; doi:10.1371/journal.pmed.1003365)
Supplement: Attachment — Submitted filename: Response to reviewers.docx [file pmed.1003365.s003.docx]

June 15^th^, 2020

Dr. Richard Turner, PhD
Senior Editor, PLOS Medicine

**Re: Responses to the reviewers’ comments on manuscript ID PMEDICINE-D-20-00878R1 entitled “Impact of providing free HIV self-testing kits on frequency of testing among men who have sex with men and their sexual partners in China: a randomized controlled trial"**

Dear Dr. Turner,

We really appreciate the reviewers’ and your valuable comments on our manuscript. We have addressed these comments one by one as follows. We have also made track changes in the revised manuscript.

| **Response to Editors:** | |
| --- | --- |
| **Comments** | **Answers** |
| 1.We ask you to make the study's primary outcome clear in the abstract, and quote the result for this outcome. Please also list the relevant secondary outcomes. | We have added primary and secondary outcomes and have included the results for these outcomes in the Abstract on Page 3, line 47-49. |
| 2. In your abstract, please quote 95% CI alongside p values. | We have added 95% CIs. |
| 3. Throughout the paper, exact p values should be quoted unless p<0.001. | We have quoted the exact *P* values unless they are <0.001. |
| 4. At line 50, and at an appropriate point in the main text, please quote the exact dates of the start and end of recruitment. | We have added the exact starting and ending dates of recruitment: “*Between April 14 and June 30, 2018, 230 MSM were recruited*” on Page 3, line 51. |
| 5. If your study was registered after recruitment had begun, please explain the reason(s) for retrospective registration. | Our study protocol was registered before participant enrollment. The date for last update of registration was on April 9, 2018, which was earlier than April 14, 2018 when the first participant was enrolled.  Our published study protocol stated that we would start participant enrollment in December 2017 (PMID: 30552280), but the actual enrollment activity was postponed. |
| 6. Please add summary demographic details for study participants to your abstract. | We have added this information to abstract on Page 3, line 51-52. |
| 7. Please quote the numbers of HIV infections in the abstract. | We have added the numbers of HIV infections in the abstract on Page 4, line 68-69. |
| 8. Please add a new final sentence to the "methods and findings" subsection of the abstract, quoting 2-3 of the study's main limitations. | We have added one major limitation in the Abstract: “*One study limitation is that the data on SBHT frequency were solely based on self-report by the participants, but self-reported HIVST frequency was validated through counting the number of testing kits provided”* on Page 4, line 69-71. |
| 9. Please begin the sentence at line 61 with "In this study, we found that ..." or similar. | We have revised this as suggested on Page 4, line 72. |
| 10. Please refer to both the attached CONSORT checklist and protocol in the methods section of your main text. | We have referred the CONSORT checklist and protocol on Page 11-12, line 190-191. |
| 11. Please reformat the reference call-out at line 300. | We have changed the format of this reference on Page 11, line 181. |
| 12. Please remove the baseline comparisons from table 1. | We have removed the baseline comparisons in Table 1. |
| 13. Please avoid claims of "the first", such as at line 423, and where necessary add "to our knowledge" or similar. | We have made these changes as suggested on Page 30, line 480. |
| 14. Please revisit your reference list to ensure that citations meet journal format. Italics should be converted into plain text. | We have checked the format and made changes on references as suggested. |
| 15. Please remove the competing interest statement from the end of the ms (this information will appear in the metadata (via the submission form) in the event of publication. | We have removed competing interest statement from the manuscript. |
| 16. Likewise, please remove the data sharing information at line 526. We note that this information appears inconsistent with that in the submission form, where you state that all relevant data are contained in the ms. PLOS' data sharing policy is that data should be presented in the ms where possible, and where this is not possible should be accessible via a public repository, for example; if a contact person is provided, this should not be an author of the article. | We have removed the data sharing information from the manuscript. All relevant data are within the manuscript and its Supporting Information files. |
| 17. Please adapt the attached CONSORT checklist so that individual items are referred to not by page or line numbers (which generally change in the event of publication) but by section (e.g., "Methods") and paragraph numbers. | We have revised as suggested. |
| Response to Reviewer #1: | |
| **Comments** | **Answers** |
| 1. Page 3 It would be better to present confidence intervals here for the differences, not just p–values. This also applies elsewhere in the paper. | We have added 95% CIs throughout the manuscript. |
| 2. Page 11 The allocation procedure presented here is not the gold standard way of doing this. Using blocks of size two is too small and using a fixed block size makes it too easy to guess the next allocation at some point. It would have been better to use sliding block sizes. Doing the whole thing with sealed envelopes is also open to possible abuse. Obviously this cannot be changed now but it is not ideal. | Thanks for your comment. We agree that the allocation was not the gold approach. However, this randomization procedure was conducted in a private room, the research assistance has received research ethical training and obeyed to the allocation procedure, so the research assistant did not know the allocation for the next participant till opening the envelope, and the next participant did not know the assignment of previous participant and therefore could not guess the next participant’ assignment. We have added this sentence in the Randomization and Masking section at Page 13, line 224-229. |
| 3. Page 15 I am afraid I did not understand the sample size determination at all. It reads as though recruitment happened in two strata: recent testers and non–recent. Was that the plan? Given the standard deviations the outcome cannot feasibly be regarded as Gaussian so what test is being considered here? | Yes, we calculated sample sizes for both subgroups separately and both were based on normal distribution assumption, as the sample sizes of articles that we referred to were reasonably large [13,22]. However, we combined two subgroups in all data analyses. We compared testing frequency between two study arms by assuming a normal distribution (Table 2 and Table 4), as Poisson distribution is approximate to normal distribution when sample size is large. Meanwhile, we also conducted data analyses assuming Poisson distribution (see new Table 3). |
| 4. Page 15 We need more detail here of the imputation model. It would also be better to cite the package using the journal reference its authors provide. https://www.jstatsoft.org/v45/i03/ seems to be what is returned by citation("mice"). | We have added the details of the imputation model on Page 17, line 304-307, and have changed the reference as suggested. |
| 5. Page 15 The decision to combine the recent and non–recent testers needs to be justified better than this. The authors have already stated they expected there to be many fewer of them earlier on the page. If they were expected to be different then at least include a two–level factor in all the models to allow for the differences. | Thanks for your suggestions. We have justified this on Page 16-17, line 295-300 as “*Per protocol the sample size was calculated for two subgroups of participants separately: recent-testers (n=184) and non-recent testers (n=26) [30]. The sample size of non-recent testers was small and did not support multivariate analysis. As non-recent testers were equally distributed in intervention and control arms, we combined non-recent testers and recent testers in all data analyses. The subgroup variable was included as a confounding factor in all multivariate models*” (also see tables 2, 3, and 4). |
| 6. Page 16 We really need a list here specifying which confounders were adjusted for. This is not mentioned in the results section as far as I can see. | We have listed confounders on Page 18, line 318-320 and line 330-332 as “*...by adjusting for potential confounders, including subgroup, age, ethnicity, residence, cohabitation, duration of living in a study city, education level, sexual orientation, marital status, occupation, and monthly personal income*”. |
| 7. Page 18 ‘92% considered themselves homosexual, 26% bisexual, and 2% heterosexual’ 92 + 26 + 2 = 100. I suspect 92 should be 72 but the authors need to check this. | Thanks for pointing out the typo. We have corrected this sentence as: “*72% considered themselves homosexual, 26% bisexual, and 2% heterosexual*” on Page 20, line 363-364. |
| 8. Pages 19 to 21, Table 1 It is illogical to test for baseline differences. The null hypothesis is that they were drawn from the same population and any differences are due to chance. But you know for sure that they are due to chance, you randomised them (Senn, 1994). | We have deleted the baseline comparisons in Table 1. |
| 9. Page 22, Table 2 Of what is this the median and quartiles? Ideally it should be of all the pairwise differences but that is not specified. | We have deleted the columns for median and quartiles in Table 2 and Table 4. |
| 10. Page 24 Why tell us the total number of partners? Would it not be more meaningful to quote some summary statistic per individual? | Thanks for your advice. We have revised to report the statistic per individual as “There was *no statistically significant difference in the mean number of sexual partners reported by MSM participants between the intervention and control arms (7.25 vs. 6.98, P=0.912)*”on Page 26, line 425-427. |
| 11. Modelling   The main outcome is a count: number of tests. The natural distribution to consider for counts is the Poisson. Rather than use rank–sum methods the use of a linear model with a log link would have enabled us to obtain easily interpretable coefficients for the ratio between the groups leading to statements that the intervention arm took X% more tests with a confidence interval from L to U. It would also be worth checking for zero–inflation relative to Poisson and over–dispersion. I suppose that an ordered categorical model might also be possible here too. If the authors really want to work on the additive scale then doing a linear model and then using bootstrapping to get the correct confidence intervals might be considered. | We have added Zero-Inflated Poisson regression analysis for analyzing the count data and checked for over-dispersion (on Page 18, line 324-333 and new Table 3). |
| 12. Adverse events   If I understand Page 27 correctly the only seroconversions occurred in the intervention arm. There were 11 of them. Using a binomial test this shows a difference at the p < 0.001 level so this is worthy of some comment. I would have thought that seroconverting counted as an serious adverse event. I see no mention of the trials steering committee or DSMB/DMEC membership in the paper but if a DMEC of which I was a member had received an interim report that a serious adverse event was split 6–0 between the arms I think alarm bells would have rung. | The study outcomes were the frequency of HIV tests among MSM participants and their sexual partners, not HIV seroconversion. However, we did ask participants to report testing results during 12 months of follow-up: 3 positive tests among MSM participants and 8 among their sexual partners. The analysis of these positive cases was not included in this manuscript.  Identifying HIV infection is the purpose of HIV testing intervention, so HIV positive testing was not considered as adverse event in this study. Adverse events related to our intervention included disclosure of homosexuality, related psychological and social impacts like discrimination, loss of job or suicide, etc. We closely monitored these events and none was reported, which was presented on Page 29, line 459-461. We provided psychological counseling service through a 24/7 hotline, and we also linked those with a HIV positive result to care, which were presented on Page 14, line 240-244. |
| Response to Reviewer #2: | |
| 1. Abstract   On line 46 there should be a space between '1' and 'year' | We have corrected it on Page 3, line 46. |
| 2. Introduction   Mentions the first 90 in the 90-90-90 targets, it is worth explaining what those targets are for those who do not know. | We have explained this on Page 8, line 112-114. |
| 3. Line 109 Would change 'homosexual transmission' to transmission between MSM. | We have revised this sentence as “*One significant characteristic of the evolving HIV epidemic in China in recent years has been the rapid increase of transmission between MSM”* on Page 8, line 120-121. |
| 4. Line 118 - I believe the evidence is conclusive that most transmissions are from those who are not yet diagnosed. Strengthen the language here, it strengthens the argument for this paper. | We have revised the sentence as: “*PLWH who don’t know their HIV status are more likely to transmit HIV than those who do”* on Page 9, line 131-132*.* |
| 5. Line 121 - there are three relevant studies here, Rodger et al 2019 and 2018   and Bavinton BR, Pinto AN, Phanuphak N, Grinsztejn B, Prestage GP, Zablotska-Manos IB, Jin F, Fairley CK, Moore R, Roth N, Bloch M. Viral suppression and HIV transmission in serodiscordant male couples: an international, prospective, observational, cohort study. The lancet HIV. 2018 Aug 1;5(8):e438-47. | Thanks for your suggestions. We have cited these three papers on Page 9, line 135. |
| 6. Line 127 Key reference for values and preferences is Figueroa et al 2015. Values and preferences on HIV self-testing for key populations. | Thank you for the suggestion. We have cited this paper on Page 9, line 141. |
| 7. Line 132 There are in fact two other relevant RCTs in China which should be mentioned   Tang W, Han L, Best J, Zhang Y, Mollan K, Kim J, Liu F, Hudgens M, Bayus B, Terris-Prestholt F, Galler S. **Crowdsourcing HIV test promotion videos: a noninferiority randomized controlled trial in China.** Clinical infectious diseases. 2016 Jun 1;62(11):1436-42.  And  Zhu X, Zhang W, Operario D, Zhao Y, Shi A, Zhang Z, Gao P, Perez A, Wang J, Zaller N, Yang C. **Effects of a mobile health intervention to promote HIV self-testing with MSM in China: a randomized controlled trial**. AIDS and behavior. 2019 Nov 1;23(11):3129-39. | These two studies were not the intervention of providing testing kits and were not similar to our study intervention. However, we have cited these two studies as they used novel methods for promoting HIV testing on Page 8, line 125-127. |
| 8. Line 148 - I would replaced 'should' with 'may'. | We have replaced this word on Page 10, line 162. |
| 9. Methods   Line 161 - says randomised clinical trial where in other sections it says randomised controlled trial. The latter is accurate. | We have revised “randomised clinical trial” to “randomised controlled trial” on Page 11, line 187. |
| 10. Line 194 - make it absolutely explicit whether the person enrolling the participants knew what the randomisation outcome would be for the next participant. | We have added a sentence: “*The research assistant enrolling the participants did not know the randomization outcome until the envelope was opened by participant*” on Page 13, line 224-226. |
| 11. Line 224 - more information is needed about this kit. Would be worth including a figure with a photo and explicitly describe if it was adapted by the manufacturer for self-use. Also mention what (if any) additional modifications the study team made. | When initiating this study, no approved HIV self-testing kits were vailable in China. But the rapid HIV testing kits could be piloted to use in the study project through CDC or community-based organizations. Thus, the rapid HIV testing kits were used in this study. Our team modified the kits for self-use by providing “*detailed electronic users’ instructions and counseling information, including 24/7 hotlines and an official WeChat study account to reach research assistants to obtain consultation on the HIVST administration and interpretation of testing results(https://mp.weixin.qq.com/s/vi9Nl-uLOeWnoJajvsq63Q)*”, which was presented on Page 14, line 240-244. |
| 12. Line 233 Outcomes   On my reading of this subsection it was not clear how these were being captured from sexual partners of participants. It is really important to clarify this as it will impact on quality assessment for systematic reviewers. | The data on HIV testing frequency among sexual partners were based on self-report by MSM participants. We have added a statement “*the secondary outcome...was indirectly reported by MSM participants*” on Page 15, line 271. In addition, we have added a limitation in the Discussion section: “*Second, the number of PHT was indirectly reported by participants because of difficulties in accessing each of their partners. This information bias may lead to under-reporting of PHT frequency in both arms, but it may not change the study result showing a significant difference between two arms”* on Page 31, line 497-501. |
| 13. 252 - make it clear in this section which (if any) outcomes were validated and how this was done. This is essential information for systematic reviewers. | The data on frequency of HIV tests among MSM participants and their partners were based on self-report of MSM participants. The self-reported number of HIVST in the intervention arm was validated by counting the number of HIVST kits provided by the study. We have added the sentence in Outcomes section: *“The self-reported number of HIVST in the intervention arm was validated by counting the number of HIVST kits provided by the study”* on Page 15, line 272-275.  We have also added two limitations in Discussion section: “*First, the data on SBHT frequency were solely based on self-report by the participants. However, self-reported HIVST frequency in the intervention arm was compared with that in the control arm by counting the number of testing kits provided by the study, and the data were consistent. This provides indirect evidence on the validity of SBHT data. Second, the number of PHT was indirectly reported by participants because of difficulties in accessing each of their partners. This information bias may lead to under-reporting of PHT frequency in both arms, but it may not change the study result showing a significant difference between two arms”* on Page 30-31, line 493-501. |
| 14. 295 - patient and public involvement. On reading this is seems to be this is formative research rather than PPI. Would be worth clarifying what the process was here and what exactly was done, what questions were asked and what modifications were made in response to this. Important to demonstrate the rigour of the process. | We agree. We have revised the whole paragraph and moved it to the start of Methods part on Page 11, line 175-183: ***“Formative research***  *During developing the RCT study protocol, we conducted formative research by interviewing* *23 MSM. Interview questions included acceptability of HIVST, preference of oral or finger-stick self-testing kits, preference for the way of delivering testing kits, and perception on partner testing. The majority (87%, 20/23) reported willingness to use HIVST, 89% (17/19) preferred finger-stick rather than oral rapid self-testing kits, all chose using express mail for delivering HIVST kits, and 87% (13/15) said it feasible to do partner testing [30]. Findings from this formative research were used to guide protocol development. Men in this interview were not involved in the study design, participant recruitment or implementation of RCT”.* |
| 15. Results   These look good and sensible to me. Worth having checked by statistician. | We have checked the results by statisticians (Brecht ML & Zheng S [we added acknowledgement to him for his counseling on statistics]) and made necessary changes as shown in the responses to the first reviewer’s comments. |
| 16. Discussions   Clear, appropriate and cautious interpretation of findings. It is important to situate these findings within the context of the other 3 RCTs in China (two of which I have provided references for earlier in this review).  Useful discussion of limitations | We have situated our research within the context of the other 3 RCTs in China (see above).  We have discussed three limitations. |
| Response to Reviewer #3: | |
| 1. The definition of sexual partners, the study did not describe the type of sexual partners, whether they are regular, casual and commercial. Giving that most MSM does have more than one partners, can a participating MSM give HIVST kits to more than one sexual partner? Based on the information in Table 3, it says nine participating MSM did not have sexual partners during 12 months of following, that is, 216 - 207 = 9. Does it mean each of the 207 MSM has exactly one sexual partner during the 12 months of follow up? | We agree with you that most MSM have more than one partner. So we compared the mean number of sexual partners between participants in the intervention group and those in the control group as “*There was no statistically significant difference in the mean number of sexual partners reported by MSM participants between the intervention and control arms (7.25 vs. 6.98, P=0.912)*” on Page 26, line 425-427.  In table 4, it means the 207 MSM has at least one sexual partner during the 12 months of follow up. |
| 2. How was the testing coverage and frequency of the sexual partners of the participating MSM measured, as in Table 3? Did the study reach the sexual partners directly or through the participating MSM to obtain the testing information? Were the test results of the sexual partners sent to the study administration in the same way as the participating MSM (that is, via WeChat, but they are not considered as study participants)? Do these sexual partners also conduct the three months questionnaires as the participating MSM? | The number of HIV tests and the relevant results for sexual partners were indirectly reported by participants because of difficulties in accessing each of their partners.  The outcome measurement was described as: “…*Both were based on self-report of MSM participants. The self-reported number of HIVST in the intervention arm was validated by counting the number of HIVST kits provided by the study”* on Page 15, line 271-274. |
| 3. Besides, I am a bit confused. In both Table 1 and Table 3, the total number of HIV tests is 2? Did you mean average? In table 3, If 67.1% received ≥1 test in 12 months, does it mean 32.9% sexual partners did not receive the test? It would be better if this information is laid out more explicitly. | To help understanding easily, we have added “(mean, SD)” or “(n, %) ” in these two tables (now Table 2 and Table 4).  Both Chinese and European guidelines recommend that individuals who are at high risk for HIV infection should be tested every 3 to 6 months. That is, they are recommended to test for HIV at least 2 times every year. So we set the cut-off value of total number of HIV tests as two in Table 2 and Table 3 to describe how many of them meet the HIV testing frequency guideline. The total number of HIV tests above 2 does not mean the average, but the cut-off score for statistical description.  For that 67.1% received ≥1 test in 12 months, it means 32.9% sexual partners did not take an HIV. |
| 4. Line 179, what is the implication of 'above 35 on the Brief Psychiatric Rating Scale.' | We have revised this sentence: “*Men were excluded if they scored above 35 on the Brief Psychiatric Rating Scale, which indicates having a psychiatric disorder”* on Page 12, line 205-207. |
| 5. Line 272-274, please identify the proportion of missing data and which indicators are missing. | We have added the sentences on Page 17, line 304-307*: “**The proportion of missing data in all four follow-up questionnaire surveys was 4.4% (38/864). Considering it is less than 5%, we assumed data missed completely at random and filled the number of HIV tests through* *multiple imputations using R software”.* |
| 6. Line 278, the study seems to separate non-recent testers and recent testers initially and then later combines them. Then what is the point of separating them in the first place? | In this manuscript, we planned to conduct multivariate analysis. “*Per protocol the sample size was calculated for two subgroups of participants separately: recent-testers (n=184) and non-recent testers (n=26) [30]. The sample size of non-recent testers was small and did not support multivariate analysis. As non-recent testers were equally distributed in intervention and control arms, we combined non-recent testers and recent testers in all data analyses. The subgroup variable was included as a confounding factor in all multivariate models*”. We justified this on Page 16-17, line 295-300. |
| 7. Line 328, the flowchart, does mean the participants are being tested at the same time as the questionnaire? | No, participants took HIV testing between two questionnaire surveys. |
| 8. Line 342, Table 2, Sexual Orientation, it seems to miss a chi-2 value? | We used a Fisher's exact test for comparing sexual orientation, so no chi-2 value was reported. As suggested by the editor, we dropped out baseline comparisons in the revised Table 1 (note: not Table 2). |
| 9. Line 418, although this approach has been demonstrated to effective, it will be largely based on the social network of MSM (one has to give the testing kit to his partner). I am not sure how it claims to be 'easily scaled-up through the extensive Chinese public health networks'? Not sure how the government network may facilitate the scale-up. | We have revised this sentence as *“This effective intervention has the potential to be easily scaled up through the increasingly expanding social and sexual networks of MSM in China”* on Page 29-30, line 474-476*.* |
