# Supplementary material for: Impact of providing free HIV self-testing kits on frequency of testing among men who have sex with men and their sexual partners in China: A randomized controlled trial
Source: PLoS Med. 2020 Oct 9;17(10):e1003365. doi: 10.1371/journal.pmed.1003365 (PMC7546488; doi:10.1371/journal.pmed.1003365)
Supplement: Attachment — Submitted filename: Response to reviewers.docx [file pmed.1003365.s005.docx]

July 29^th^, 2020

Dr. Richard Turner, PhD
Senior Editor, PLOS Medicine

**Re: Responses to the reviewers’ comments on manuscript ID PMEDICINE-D-20-00878R2 entitled “Impact of providing free HIV self-testing kits on frequency of testing among men who have sex with men and their sexual partners in China: a randomized controlled trial"**

Dear Dr. Turner,

We really appreciate the reviewers’ and your valuable comments on our manuscript. We have addressed these comments one by one as follows. We have also made track changes in the revised manuscript.

| **Response to the Editor:** | |
| --- | --- |
| **Comments** | **Answers** |
| 1. At lines 36 and 83, we suggest substituting "growing" for "increasing". | We have changed the word at line 27 (page 2) and line 76 (page 5). |
| 2. Please mention in the abstract that no adverse events were reported. | We have added a sentence about it at line 60 (page 3). |
| 3. Please quote one further study limitation around line 70. | We have added one more limitation at lines 63-64 (Page 3). |
| 4. Please remove the information on funding at the end of the main text - this information will appear in the article metadata in the event of publication, via entries in the submission form. | We have removed this information. |
| Response to Statistician Reviewer #1: | |
| **Comments** | **Answers** |
| 1. I said: *I am afraid I did not understand the sample size determination at all. It reads as though recruitment happened in two strata: recent testers and non–recent. Was that the plan? Given the standard deviations the outcome cannot feasibly be regarded as Gaussian so what test is being considered here?*   As far as I can see all the details of the sample size determination have now be removed completely from the manuscript. This is a mistake, the sample size determination is a key part of the reporting of a clinical trial and must be included. | Yes, we removed the sample size calculation information in the previous revision, as we referred it to published study protocol. Our response to this comment in the previous revision was: “Yes, we calculated sample sizes for both subgroups separately and both were based on normal distribution assumption, as the sample sizes of articles that we referred to were reasonably large [13,22]. However, we combined two subgroups in all data analyses. We compared testing frequency between two study arms by assuming a normal distribution (Table 2 and Table 4), as Poisson distribution is approximate to normal distribution when sample size is large. Meanwhile, we also conducted data analyses assuming Poisson distribution (see new Table 3).”  Now we have restored and edited the sample size calculation in the updated version: “*Sample sizes were calculated for recent and non-recent testers separately based on normal distribution of HIV testing frequencies. G-power V.3.0 was used to calculate the sample size considering a 5% significance level [34], 80% power, and a one-tailed test. Recommendation from guidelines suggested two to four HIV tests per year for individuals at high risk [8,9]. Given the missing rate of 20%, we conservatively estimated 184 recent-testers were needed to detect the increase from 1.35 to 2 (SD 1.61) HIV tests per year [13], and 26 non-recent testers were needed to detect the increase from 0.2 to 1 (SD 0.7) HIV test per year [22]*” (lines 289-296, pages 15-16). |
| 2. I said: *The decision to combine the recent and non–recent testers needs to be justified better than this. The authors have already stated they expected there to be many fewer of them earlier on the page. If they were expected to be different then at least include a two–level factor in all the models to allow for the differences.*   However despite what the authors say in the rebuttal Table 2 makes it clear that sub–group was not included in the model. This is because the authors have used variable selection although this is not reported in the methods  section where it should be. Selecting a subset of variables in a way driven by the data leads to a model which is unlikely to replicate (Babyak, 2004). Step–wise methods, if those was what the authors have used, give biased coefficients, goodness of fit is exaggerated, p-values and confidence intervals lose their normal meaning, they have problems in the presence of collinearity,  they often fail to replicate on new datasets (although they are rarely tested in that way) (Copas, 1983; Hurvich and Tsai, 1990; Miller, 1990; Derksen and Keselman, 1992).  The authors need to fit a full model using all the theoretically specified confounders. They should also make sure the methods section clearly specifies what was to be done. | We have revised Table 2 and Table 4. We used full models adjusting for all potential confounders, including subgroup, as did for Table 3. |
| 3. I said: *If I understand Page 27 correctly the only seroconversions occurred in the intervention arm. There were 11 of them. Using a binomial test this shows a difference at the p < 0.001 level so this is worthy of some comment. I would have thought that seroconverting counted as an serious adverse event. I see no mention of the trials steering committee or DSMB/DMEC membership in the paper but if a DMEC of which I was a member had received an interim report that a serious adverse event was split 6–0 between the arms I think alarm bells would have rung.*   The authors in their rebuttal do not feel that seroconversion would be an adverse event. So if everybody in the intervention group had increased their  frequency of testing but also seroconverted that would be a success? That seems odd to me. | One purpose of HIV testing is to get early diagnosis of HIV infection so the infected individuals could take preventive measure for further transmission or get early treatment to prevent disease progression. In this sense, HIV seroconversion is not considered as adverse event.  For HIV testing intervention trial like this, potential adverse events may include accidentally disclose HIV positive results to participant’s family members, coworkers resulting in loss of jobs or suicide. In our study, there was no adverse event.  The reasons for all 11 HIV positives being reported in the intervention arm may include: First, of 11 positives reported in the intervention arm, 3 were from participants and were considered as seroconversions (as they were baseline negative), while 8 were from their sexual partners , and they could be just positive (not necessarily new seroconversions). Second, participants in the intervention arm reported HIV testing results to the research assistant to get refill of HIVST kits, so they had motivation to report testing results, while participants in the control arm did not have this motivation, so the control participants might underreport HIV positives. Third, a higher proportion of participants in the intervention arm took HIV testing and therefore they were more likely to report HIV positive results than the control: HIV testing rates at 3-month (79.1% vs. 44.3%; *P*<0.001), 6-month (70.9% vs. 49.1%; *P*=0.001), 9-month (69.1% vs. 39.6%; *P*<0.001) and 12-month follow-ups (66.4% vs. 33.6%; *P*<0.001).  However, HIV seroconversion was not the study outcome, as this study was not statistically powered for assessing the difference of HIV seroconversions between two study arms. |
| 4. The authors state that all the data is either in the manuscript or in supplementary files. I did not find it there. | We have clarified this in the system as “*As the data contain potentially identifying or sensitive patient information, they cannot be made publicly available. The data of the current study can be available through request from researchers by contacting the corresponding author Dr. Xianhong Li (xianhong_li@csu.edu.cn).*” |
| Response to Reviewer #2: | |
| 1. It is great to see a clearer description of the formative research and the findings. I would note that although you conducted a qualitative study, you have reported these as quantitative outcomes. It would be more useful to have a very brief description of the themes which emerged in interviews (e.g. reasons why HIVST could increase uptake, why MSM preferred finger stick tests) and a description of how these findings were used in protocol design. If indeed this was a survey study rather than an interview study then better to change the language to reflect that. | This formative research was a survey. We have changed the word “interview” to “survey” in this paragraph. |
| Response to Reviewer #3: | |
| 1. I am satisfied with the edits the authors made, no further comments from me. | We greatly appreciate your time for reviewing this paper. |

Xianhong Li, PhD
