# Supplementary material for: Impact of providing free HIV self-testing kits on frequency of testing among men who have sex with men and their sexual partners in China: A randomized controlled trial
Source: PLoS Med. 2020 Oct 9;17(10):e1003365. doi: 10.1371/journal.pmed.1003365 (PMC7546488; doi:10.1371/journal.pmed.1003365)
Supplement: Attachment — Submitted filename: Response to editors.docx [file pmed.1003365.s006.docx]

August 24^th^, 2020

Jessica Hyde
Publications Assistant

**Re: Responses to the editors’ comments on manuscript ID PMEDICINE-D-20-00878R4 entitled “Impact of providing free HIV self-testing kits on frequency of testing among men who have sex with men and their sexual partners in China: a randomized controlled trial"**

Dear Hyde,

We really appreciate your valuable comments on our manuscript. We have addressed each of the comments on the table below. We have also made track changes in the revised manuscript.

| **Response to the Publications Assistant:** | |
| --- | --- |
| **Comments** | **Answers** |
| 1. We have noted that you have the following present in your manuscript: List of keywords/ Author Contributions   We have removed these section from your manuscript file as this information is automatically pulled from the submission form. Please make sure that the information in the submission form is correct. | Thanks. We included correct information in the submission form. |
| 2. Please ensure that all Supporting Information files are clearly labeled in the file inventory. i.e. S1_Fig.tif, S1_Table.xlsx, S1_Text.doc, S1_Data.xlsx, S1_PRISMA_Checklist.docx, etc.   Each file type should be numbered starting at 1. Please also make sure that the file description matches the file name (e.g. S1_Fig.tif is listed as S1 Fig). | We have changed the labelling of supporting information files as suggested. |
| 3. Please ensure that all main figures are referenced in the manuscript as Fig 1, Fig 2, etc. (including capitalization), rather than Figure 1, Figure 2, etc. Please note, however, that the file names themselves must not include the space, i.e. Fig1.tif, Fig2.tif. Please ensure that all main figure files are clearly labeled as such in the File Inventory. | We have double checked and all are correctly referenced. |
| 4. Thank you for adding effect sizes. However, with apologies for any misunderstanding that we have created, we feel that "effect size" is too general a term to use in a medical journal. Please confer with your trial statistician and let us know if you disagree with the following requests, or if this contradicts information in the trial protocol/statistlcal analysis plan.   We have checked in with a statistical advisor, and ask that you adapt the presentation to substitute "standardized mean difference (SMD)" for effect size at line 39 in the abstract ("The effect size for the primary and secondary outcomes was evaluated as the standardized mean difference (SMD) in testing frequency ...") or similar, also substituting "SMD" for "ES" thereafter throughout the ms. | We agree with your suggestion. We have changed the definition in Abstract and Methods section, and have replaced SMD for ES throughout the manuscript. |
| 5. Please make similar changes in the author summary (starting at line 93) and methods section (starting at line 319). | We have revised as suggested at author summary, methods section, results section, table 2 and table 4. |
| 6. Please make that "Trial" at line 72. | This has been revised. Thanks for your advice. |
| 7. In the header of table 2, please convert "Odd" to "Odds" and substitute "Standardized mean difference" for "Effect size". | These have been revised as suggested. |
| 8. Please also adapt the CONSORT checklist to note that standardized mean differences were calculated. | This has been revised. Thanks for your thoughtful suggestions. |

Xianhong Li, PhD
